# Histone Chaperone Paralogs Have Redundant, Cooperative, and Divergent Functions in Yeast

**DOI:** 10.1534/genetics.119.302235

**Published:** 2019-10-11

**Authors:** Neda Savic, Shawn P. Shortill, Misha Bilenky, Joseph M. Dobbs, David Dilworth, Martin Hirst, Christopher J. Nelson

**Affiliations:** * Department Biochemistry and Microbiology, University of Victoria, BC V8W 3P6, Canada; † BC Cancer Agency Genome Sciences Centre and the Department of Microbiology & Immunology, Michael Smith Laboratories, University of British Columbia, Vancouver, BC V6T 1Z3, Canada

**Keywords:** chromatin, paralog, histone chaperone, genetic interactions, nucleolus

## Abstract

Gene duplications increase organismal robustness by providing freedom for gene divergence or by increasing gene dosage. The yeast histone chaperones Fpr3 and Fpr4 are paralogs that can assemble nucleosomes *in vitro*; however, the genomic locations they target and their functional relationship is poorly understood. We refined the yeast synthetic genetic array approach to enable the functional dissection of gene paralogs. Applying this method to Fpr3 and Fpr4 uncovered redundant, cooperative, and divergent functions. While Fpr3 is uniquely involved in chromosome segregation, Fpr3 and Fpr4 cooperate to regulate genes involved in polyphosphate metabolism and ribosome biogenesis. We find that the TRAMP5 RNA exosome is critical for fitness in Δ*fpr3*Δ*fpr4* yeast and leverage this information to identify an important role for Fpr4 at the 5′ ends of protein coding genes. Additionally, Fpr4 and TRAMP5 negatively regulate RNAs from the nontranscribed spacers of ribosomal DNA. Yeast lacking Fpr3 and Fpr4 exhibit a genome instability phenotype at the ribosomal DNA, which implies that these histone chaperones regulate chromatin structure and DNA access at this location. Taken together. we provide genetic and transcriptomic evidence that Fpr3 and Fpr4 operate separately, cooperatively, and redundantly to regulate a variety of chromatin environments.

GENE duplication events play an important role both in driving protein evolution and in providing a mechanism for ensuring the robustness of biological systems. Since the earliest observations of duplications on chromosomes (Darlington and Moffett 1930; Bridges 1936) and redundant genes (Kataoka *et al.* 1984; Basson *et al.* 1986), models implicating gene duplication events as complex drivers of evolution have been proposed (Ohno 1970; Hughes 1994; Force *et al.* 1999; Francino 2005; Innan and Kondrashov 2010). Evolutionary forces can favor the retention of redundant genes for dosage reasons; for example, identical copies of histone and ribosomal genes are present in most eukaryotes. Alternately, duplicated genes provide an opportunity for functional divergence of gene pairs, or paralogs, over time.

The *FPR3* and *FPR4* genes encode two *Saccharomyces cerevisiae* paralogs (Benton *et al.* 1994; Manning-Krieg *et al.* 1994; Shan *et al.* 1994; Dolinski *et al.* 1997) derived from a distant ancestral gene (Wolfe and Shields 1997; Kellis *et al.* 2004; Pemberton 2006). They code for highly similar proteins (58% identical and 72% similar in amino acid residues) with acidic N-terminal nucleoplasmin-like histone chaperone and C-terminal FK506-binding (FKBP) peptidyl-prolyl isomerase domains (Kuzuhara and Horikoshi 2004; Xiao *et al.* 2006; Park *et al.* 2014) (Figure 1A). Both proteins localize to the nucleus and are enriched in the nucleolus (Benton *et al.* 1994; Manning-Krieg *et al.* 1994; Shan *et al.* 1994; Huh *et al.* 2003). Notably, Fpr3 and Fpr4 interact with each other and share some common physical interactors (Krogan *et al.* 2006), including histones (Shan *et al.* 1994; Nelson *et al.* 2006; Xiao *et al.* 2006) and the Nop53 ribosome biogenesis factor (Sydorskyy *et al.* 2005). Additionally, both *FPR3* and *FPR4* are multicopy suppressors of temperature sensitivity and mating defects resulting from the absence of the Tom1 E3 ubiquitin ligase (Utsugi *et al.* 1999; Davey *et al.* 2000), and both Fpr3 and Fpr4 are required for the degradation of the centromeric histone H3 variant Cse4 (Ohkuni *et al.* 2014). Therefore, there is good evidence that Fpr3 and Fpr4 cooperate.

**Figure 1:**
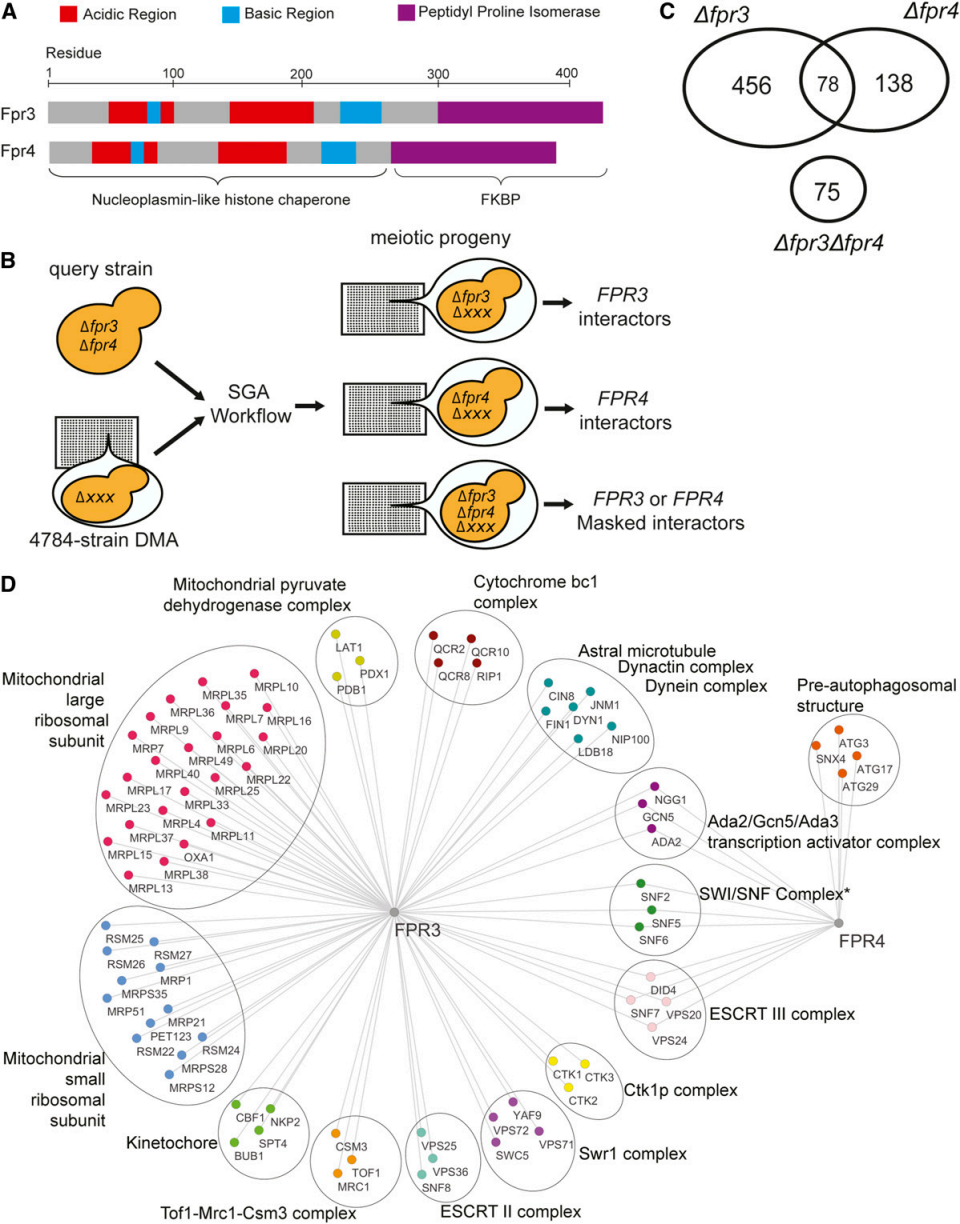
Fpr3 and Fpr4 have separate, cooperative, and redundant functions. (A) Domain architectures of Fpr3 and Fpr4. Both proteins have an N-terminal nucleoplasmin-like domain with characteristic patches of acidic and basic residues, and a C-terminal FK506-binding (FKBP) peptidyl prolyl isomerase domain. (B) Schematic illustrating modified paralog-SGA workflow. Spores from a single cross of the double deletion ∆*fpr3*∆*fpr4* query to the 4784 strain DMA are manipulated to generate three separate sets of meiotic progeny for interactome analysis. The query strain also harbored an episomal *URA3* plasmid with a functional *FPR4* gene to avoid the slow growth phenotype of Δ*fpr3*Δ*fpr4* dual deletion, and its vulnerability to suppressor mutations. This plasmid was selected for (for *FPR3* interactors) or against (for *FPR4* interactors) in the last step of the screen. (C) Venn diagram illustrating shared and unique negative genetic interactions from ∆*fpr3* and ∆*fpr4* paralog-SGA screens. The number of negative genetic interactions only detectable in double deletion ∆*fpr3*∆*fpr4* mutants is represented below. (D) Network illustrating complex related ontologies enriched among unique and shared negative genetic interactors of *FPR3* and *FPR4*. * *SNF2*, *SNF5*, and *SNF6* were identified as hits in the *FPR4* screen only, but displayed a synthetically sick phenotype with both ∆*fpr3* and ∆*fpr4* mutations in confirmatory spotting assays (not shown).

There is also evidence that these paralogs have separate functions. Fpr3 has been identified as a regulator of chromosome dynamics at mitotic and meiotic centromeres. During meiosis, Fpr3 enhances recombination checkpoint delay (Hochwagen *et al.* 2005) and prevents meiotic chromosome synapsis initiation at centromeres (Macqueen and Roeder 2009). To our knowledge, no reports describe similar data for Fpr4. Thus, Fpr3 may have functionally diverged. By contrast, Fpr4 can silence expression of a reporter at ribosomal DNA (rDNA) (Kuzuhara and Horikoshi 2004), but the degree to which Fpr3 regulates rDNA has not been described. Additionally, Fpr4 is involved in transcription induction kinetics through the isomerization of prolines on the amino tails of histones H3 and H4 (Nelson *et al.* 2006). Finally, microarray gene expression analysis of Δ*fpr3* and Δ*fpr4* yeast identified small changes in partially overlapping sets of mRNAs (up to fourfold changes in 385 and 161 genes, respectively) (Park *et al.* 2014).

Loss-of-function phenotypes and genetic interactions usually provide insight into gene function. For example, the *ASF1* and *RTT106* genes, encoding histone chaperones, display clear chromatin-related genetic interactions in synthetic genetic array (SGA) screens (Costanzo *et al.* 2010, 2016). We noted that the genetic interactomes of *FPR3* and *FPR4* contained few chromatin-related hits (Collins *et al.* 2007; Costanzo *et al.* 2010, 2016; Stirling *et al.* 2011; Milliman *et al.* 2012) and hypothesized that the high similarity of these paralogs could render them semiredundant, masking their genetic interactions.

Here, through a set of comprehensive genetic interaction screens designed for paralogs and a series of RNA-sequencing (RNA-seq) transcriptome surveys, we demonstrate that Fpr3 and Fpr4 operate separately, cooperatively, and redundantly. Unique genetic interaction profiles and differentially expressed genes demonstrate that these histone chaperones are not equivalent; for example, Fpr3 appears uniquely involved in chromosome segregation. By contrast, shared genetic interactions of *FPR3* and *FPR4* with the SWI/SNF and ADA chromatin regulators predicted that Fpr3 and Fpr4 cooperate to regulate genes. The identification of polyphosphate metabolism and ribosome biogenesis genes as Fpr3/4 targets confirms this prediction. We find that the TRAMP5 RNA exosome becomes critical for fitness in Δ*fpr3*Δ*fpr4* yeast, and leverage this information to perform a sensitized survey for Fpr4-regulated genomic loci. This strategy identified an important role for Fpr4 at the 5′ ends of protein coding genes as well as at the nontranscribed spacer regions of rDNA. Finally, we show that yeast lacking Fpr3 and Fpr4 exhibit a genome instability phenotype at the rDNA, implying that these histone chaperones regulate chromatin structure at these regions. Taken together we provide genetic and transcriptomic evidence that Fpr3 and Fpr4 operate separately, cooperatively, and redundantly to regulate a variety of chromatin environments.

## Materials and Methods

### Yeast strains and plasmids

Yeast strains used in this study are described in Supplementary Material, Appendix 5. Strains in the *MAT***a** nonessential yeast deletion mutant array (DMA) collection used for the SGA analysis are all isogenic to BY4741 and were purchased from Thermo Fisher Dharmacon. The plasmid rescued double genomic deletion ∆*fpr3*∆*fpr4* SGA query strain (YNS 35) was created in a Y7092 genetic background as follows. The endogenous *FPR4* locus on a Y7092 wild-type strain was replaced with a nourseothricin resistance (*MX4-NATR*) PCR product deletion module. The resulting single-gene ∆*fpr4* deletion mutant was subsequently transformed with prs316 FPR4: a single-copy, *URA3*-marked shuttle vector carrying an untagged, full-length copy of the *FPR4* open reading frame with endogenous promoter and terminator [originally described in Nelson *et al.* (2006)]. The endogenous *FPR3* locus on this plasmid-rescued ∆*fpr4* deletion mutant was subsequently replaced with a *LEU2* PCR product deletion module.

Triple deletion mutants ∆*rrp6*∆*fpr3*∆*fpr4* and ∆*trf5*∆*fpr3*∆*fpr4* and their corresponding mixed-population total haploid meiotic progeny controls used in the validating growth curves were generated from the SGA cross (see below).

Single-gene deletion mutants of ∆*fpr3*, ∆*fpr4*, and ∆*sir2* used for the RNA-seq are all isogenic to BY4741 and were either purchased from open biosystems or taken from the yeast DMA (purchased from Thermo Fisher Dharmacon). The isogenic double deletion ∆*fpr3*∆*fpr4* mutant was constructed from the open biosystems ∆*fpr3* single-gene deletion mutant by replacing the endogenous *FPR4* locus with a nourseothricin resistance (*MX4-NATR*) PCR product deletion module. The *FPR4*(∆*fpr3*∆*trf5**)* and ∆*fpr3*∆*fpr4*∆*trf5* isogenic strains and their corresponding total haploid mixed-population controls were generated from the SGA cross (see below).

### SGA analysis

SGA analysis was performed using a Singer Instruments ROTOR microbial arraying robot as previously described (Tong and Boone 2006), with the following modifications. The *MAT***a**/α diploid zygotes resulting from the query strain DMA cross were pinned onto diploid selective YPD + G418/clonNAT plates a total of two times for greater selection against any residual haploids. Sporulation was carried out at room temperature for 14 days. Spores were pinned onto *MAT***a** selective germination media for two rounds of selection as previously described (Tong and Boone 2006).

The resulting *MAT***a** progeny were subsequently replica plated onto four kinds of selective media: control media selective for the total haploid meiotic progeny population (SD media lacking histidine, arginine, and lysine, and containing canavanine and thialysine both at a final concentration of 50 mg/liter, and G418 at a final concentration of 200 mg/liter), media selective for ∆*xxx*∆*fpr3* haploid meiotic progeny (SD media lacking histidine, arginine, lysine, leucine, and uracil, and containing canavanine and thialysine both at a final concentration of 50 mg/liter, G418 and clonNAT both at a final concentration of 200 mg/liter), media selective for ∆*xxx*∆*fpr4* haploid meiotic progeny (SD media lacking histidine, arginine, and lysine, and containing canavanine and thialysine both at a final concentration of 50 mg/liter, G418 and clonNAT both at a final concentration of 200 mg/liter, and 5-fluoroorotic acid (5-FOA) at a final concentration of 1000 mg/liter), and finally, media selective for ∆*xxx*∆*fpr3*∆*fpr4* haploid meiotic progeny (SD media lacking histidine, arginine, lysine, and leucine, and containing canavanine and thialysine both at a final concentration of 50 mg/liter, G418 and clonNAT both at a final concentration of 200 mg/liter, and 5-FOA at a final concentration of 1000 mg/liter). Plates were incubated at 30° for 24 hr and were then expanded into triplicate and incubated for an additional 24 hr at 30°.

Images of each plate were scanned and subsequently processed using the Balony image analysis software package as previously described (Young and Loewen 2013). In brief, pixel area occupied by each colony was measured to determine colony size. Progeny fitness was then scored as follows. The ratio of each double (∆*xxx*∆*fpr3*, ∆*xxx*∆*fpr4*) and triple (∆*xxx*∆*fpr3*∆*fpr4*) mutant colony size relative to its corresponding total haploid meiotic progeny control colony was determined. Ratio cut-off thresholds were estimated automatically by the software by extrapolating the central linear portion of the ratio distributions and finding the *y*-intercepts at either ends of the *x*-axis. Genetic interactions were identified using the automatically estimated upper and lower cut-off thresholds and default Balony hit parameters (*i.e.*, reproducibility in 3/3 sets and *P*-values < 0.05) (a complete list of all genetic interactions generated from each data set is presented in Appendix 1).

### SGA data processing

Unique, common, and masked synthetic sick/lethal interactors were identified as follows. First, duplicate genes in the lists of hits from each data set were removed. The three lists of hits were then compared to each other. The ∆*fpr3* and ∆*fpr4* screens were compared to identify unique and common interactors. Genes uniquely present in the ∆*fpr3*∆*fpr4* double mutant screens were defined as masked interactors. Unique, common, and masked suppressor interactors were identified the same way.

The lists of unique, common, and masked synthetic sick/lethal and suppressor genetic interactors were subsequently analyzed using the web based FunSpec bioinformatics tool (http://funspec.med.utoronto.ca/, Dec 2017). The analysis was performed using a *P*-value cut-off score of 0.01, and without Bonferroni correction. A full list of the ontologies uncovered and their corresponding *P*-values are presented in Appendix 2. Networks illustrating the unique and common complex related genetic interactions were drawn using the Cytoscape software platform (http://www.cytoscape.org/).

### Growth curves

Growth curves to validate the synthetic sickness phenotypes were carried out as follows. Colonies generated from the SGA assay corresponding to each triple mutant of interest and its respective control colony were isolated and validated for correct genotype by PCR. Confirmed strain isolates were then resuspended in fresh YPD media, normalized to an OD_600_ of 0.2 and distributed into triplicate wells of a 24-well cell culture plate. Plates were subsequently grown for 16 hr at 30° in a shaking plate reader. Readings of OD_600_ were taken every 30 min.

### RNA-seq library preparation and sequencing

Single colony isolates of each strain were grown to midlog phase in 50 ml of liquid YPD media. Samples were then pelleted and washed once with sterile water before being flash frozen in liquid nitrogen and stored for 16 hr at −80°. Samples were thawed on ice, and RNA was extracted using a phenol freeze-based approach as previously described (Schmitt *et al.* 1990). The extracted RNA was subsequently treated with RNase-free DNase I (Thermo Fisher Scientific).

RNA samples were processed and sequenced at the BC Cancer Agency Michael Smith Genome Sciences Centre following standard operating protocols. Briefly, total RNA samples were ribo-depleted using the Ribo-Zero Gold rRNA Removal Kit (Yeast) (Illumina, San Diego, CA) and analyzed on an Agilent 2100 Bioanalyzer using Agilent 6000 RNA Nano Kit (Agilent Technologies, Santa Clara, CA). Complementary DNA (cDNA) was generated using the Superscript Double-Stranded cDNA Synthesis kit (Thermo Fisher) and 100 bp paired-end libraries were prepared using the Paired-End Sample Prep Kit (Illumina).

### Processing of sequencing data

Sequenced paired-end reads were aligned to the sacCer3 reference genome (https://www.ncbi.nlm.nih.gov/assembly/GCF_000146045.2/) using the BWA aligner (Li and Durbin 2010) (version 0.6.1-r104-tpx). We observed that out of 5110 *S. cerevisiae* genes annotated in Ensembl v.90, only 267 are spliced with 251 having one intron. Therefore, we considered genomic alignment of RNA-seq reads as a good approximation for the yeast transcriptome analysis. For every library a total of ∼1.5–2M reads were sequenced, of which ∼75–95% of reads were aligned.

To quantify gene expression, we filtered reads that aligned to multiple locations (and therefore cannot be placed unambiguously) by applying a BWA mapping quality threshold of five. We further collapsed fragments that were duplicated (only counting a single copy of a read pair if a number of pairs with the same coordinates was sequenced) and removed chastity failed reads, considering only reads that were properly paired. Postprocessing was performed using the “pysam” application for python (https://github.com/pysam-developers/pysam). The alignment statistics were calculated using the “sambamba” tool v.0.5.5 5 (Tarasov *et al.* 2015).

We considered cDNA fragment lengths distributions as well as genome-wide distributions of read coverage (data not shown) to ensure that these characteristics are similar for the pairs of data sets in the differential gene expression (DE) analysis. Genome-wide pair-ended fragment coverage profiles for both strands were generated, as well as read counts for every gene for further DE analysis.

The reads per kilobase per million values were calculated for every gene, and DE analysis was performed using the DEfine algorithm (M. Bilenky, unpublished data). First, the chi-squared *P*-value was estimated for every gene, under the null hypothesis that the gene is not differentially expressed between two data sets. The Benjamini–Hochberg false discovery rate control procedure was applied (false discovery rate = 0.05) to find a *P*-value threshold . To further reduce noise, we only considered genes with a fold change between reads per kilobase per million values of > 1.5, and required a minimal number of aligned reads of > 5 per gene. Only reads aligned to the proper strand were considered in the DE analysis.

In addition to the standard DE analysis, where gene expression quantification was done by counting reads falling into the gene boundaries, we considered a model-independent approach by calculating read counts in every 175-bp-long bin genome-wide (for both strands), and performed DE analysis between bins (with the same approach we used for genes, see above). After defining the DE bins, we overlapped their locations with gene coordinates to determine DE genes. This second approach also provided a list of potential differential gene–expressed intergenic regions. A full list of the DE genes is presented in Appendix 3.

### Quantitative real-time PCR validation of DE transcripts

Total RNA was prepared from single colony isolates of each strain grown to midlog phase in 50 ml of liquid YPD media using a phenol freeze-based approach as previously described (Schmitt *et al.* 1990). The extracted RNA was subsequently treated with RNase- free DNase I (Thermo Fisher Scientific) and cDNA was prepared using a High-Capacity cDNA Reverse Transcription Kit (Applied Biosystems). Quantitative real time PCR was performed using the Maxima SYBR Green qPCR Master Mix (Thermo Scientific) and the forward and reverse primers are listed in Appendix 6. Experimental gene Ct values were normalized to the mean Ct values of two housekeeping gene normalizers: *TCM1* and *GPD1*.

### Ontology analysis of DE genes

Ontologies associated with differentially expressed genes or genetic interactions were identified using the web-based FunSpec bioinformatics tool (http://funspec.med.utoronto.ca/, Dec 2018). The analysis was performed on genes displaying a fold change of ≥1.3, using a *P*-value cut-off score of 0.001, and with Bonferroni correction. A full list of the ontologies uncovered and their corresponding *P*-values is presented in Appendix 4.

### Averaged gene read maps

Universal gene coverage profiles were generated as follows: we first crated cDNA fragment coverage profiles genome-wide for both strands using all aligned read pairs. Next, we selected profiles for individual genes and scaled them to 100 units and normalized by the total gene coverage. After that, we agglomerated all scaled and normalized gene coverage profiles together. When doing this, the profiles for genes on the negative strand were inverted (in other words, we always agglomerated profiles from 5′ to 3′ of the gene).

### rDNA reporter propagation assays

The URA^+^ status of each reporter containing strain was first confirmed by growth on SD media lacking uracil. Saturated overnights were then prepared from single colony isolates of each confirmed strain in liquid YPD media. Cultures were prepared from the overnights in 50 ml YPD media and grown at 30° to midlog phase. Cells were subsequently collected, washed once, resuspended in sterile deionized water, and normalized to an OD_600_ = 0.5. Normalized cell suspensions were subsequently diluted 10-fold and 250 µl of each dilution was plated on 25 ml SD 5-FOA plates. Plates were incubated at 30° for 16 hr. A total of 96 well-isolated colonies were randomly picked from each 5-FOA plate using the Genetix QPix-2 colony picking robot, and deposited onto nonselective solid YPD plates. Plates were incubated for 5 days at 30°. All 96 colonies on each YPD plate were then replica-plated onto SD complete control media and SD media lacking uracil, and incubated for 5 days at 30° before being imaged.

### Data availability statement

Appendix 1 contains lists of all genetic interactions detected in this study. Appendix 2 contains the gene ontology analysis of genetic interactions. Appendix 3 contains lists of all differentially expressed genes detected in this study. Appendix 4 contains the gene ontology analysis of differentially expressed genes. RNA-seq data are deposited in the Gene Expression Omnibus Repository (accession number GSE134075). All yeast strains and primers used in this study are listed in Appendices 5 and 6, respectively. Supplemental material available at figshare: https://doi.org/10.25386/genetics.9911312.

## Results

### Genetic interactions reveal separate, cooperative, and redundant functions of *FPR3* and *FPR4*

Since Δ*fpr3* and Δ*fpr4* yeast are viable but double Δ*fpr3*Δ*fpr4* mutants display a synthetic sick phenotype (Dolinski *et al.* 1997; Costanzo *et al.* 2010), we reasoned that partial redundancy may be masking genetic interactions. To address this and determine the biological processes sensitive to these histone chaperones, we performed a modified SGA screen designed to dissect functional redundancy of gene paralogs (Figure 1B, see *Materials and Methods*). To this end, we crossed a dual-query Δ*fpr3*Δ*fpr4* double mutant strain to the 4784 strain nonessential yeast DMA, so that the fitness of all double (Δ*fpr3*Δ*xxx and* Δ*fpr4*Δ*xxx)* and triple (Δ*fpr3*Δ*fpr4*Δ*xxx)* mutant meiotic progeny could be measured in parallel. The query strain also harbored an episomal *URA3* plasmid with a functional *FPR4* gene to avoid the slow growth phenotype of Δ*fpr3*Δ*fpr4* dual deletion yeast, and its vulnerability to suppressor mutations. This plasmid was maintained until the final step of the screen, when counterselection with 5′FOA created the *fpr4* null status. Using standard selection methods, the spores of this single cross were manipulated to generate three separate SGA screens that identified all genetic interactions with Δ*fpr3*, Δ*fpr4*, and genes whose disruption affected the fitness of yeast lacking both Δ*fpr3*Δ*fpr4*.

We identified 456 and 138 genetic interactors that were unique to either *FPR3* or *FPR4*, respectively, revealing that these paralogs are not equivalent (Figure 1C, top). An additional 78 genes interacted with both *FPR3* and *FPR4*, implying that there are specific contexts of paralog cooperativity; that is, situations where both histone chaperones are required for function. We also uncovered 75 masked interactors, defined as genes whose deletion only affects the fitness Δ*fpr3*Δ*fpr4* yeast (Figure 1C, bottom). These genes highlight processes where paralog function is redundant. The complete list of these genes and a gene ontology analysis are provided in Appendices 1 and 2, respectively.

*FPR3* genetic interactors fall into a diverse collection of protein complex ontologies, including members of the large and small mitochondrial ribosomal subunits (*P* < 10^−14^ and *P* = 7.49 × 10^−7^, respectively), the mitochondrial pyruvate dehydrogenase complex (*P* = 1.16 × 10^−3^), the cytochrome bc1 complex (*P* = 3.11 × 10^−3^), and components of the ESCRT II endosomal sorting complex (*P* = 3.06 × 10^−4^) (Figure 1D). We also identified all three components of the Ctk1 kinase complex (*P* = 3.06 × 10^−4^) and four components of the Swr1 chromatin remodeler (*P* = 9.00 × 10^−3^), supporting at least some potential chromatin-centric roles of Fpr3. Most notably, we uncovered complexes involved in chromosome segregation such as the astral microtubule (*P* = 6.48 × 10^−6^), kinetochore (*P* = 1.14 × 10^−4^), and the Mrc1/Csm3/Tof1 complex (*P* = 3.06 × 10^−4^) as genetic interactors unique to Fpr3, and not Fpr4. These systems-level data support reports indicating that Fpr3, but not Fpr4, regulates mitotic and meiotic chromosome dynamics, including those associated with centromeres (Hochwagen *et al.* 2005; Macqueen and Roeder 2009; Ohkuni *et al.* 2014). Although we identified 138 *FPR4*-specific genetic interactions, they fall into limited ontologically related protein complex categories. Several genes coding for components of the preautophagosome and associated with the process of mitochondrion degradation (*P* = 2.89 × 10^−3^) were the exception, but the relationship between Fpr4 and this process is not clear. Taken together, the number and nature of negative genetic interactions from single-query screens suggest that Fpr4 cannot fulfill many of the biological functions of Fpr3, particularly those in chromosome dynamics and mitochondrial ribosome biology. However, Fpr3 might be able to substitute for Fpr4 (see below).

Shared genetic interactions would be expected if both paralogs were required for the efficient execution of a biological process. Among genetic interactors common to both *FPR3* and *FPR4* are genes coding for the ESCRT III complex (*P* = 6.05 × 10^−6^), which functions in endosomal sorting; the Ada2/Gcn5/Ada3 histone acetyltransferase (*P* = 1.50 × 10^−5^); and the ATP-dependent SWI/SNF chromatin remodeler (Figure 1D). Shared genetic interactions with the SWI/SNF remodeler were confirmed using spotting assays (data not shown). The proposed cooperation of Fpr3 and Fpr4 is supported by the fact these proteins copurify (Krogan *et al.* 2006) and, like nucleoplasmin, have the intrinsic propensity to form oligomers (Dutta *et al.* 2001; Edlich-Muth *et al.* 2015; Koztowska *et al.* 2017). Thus, these shared genetic interactions with known chromatin regulatory complexes support published protein complex data and indicate that Fpr3 and Fpr4 likely cooperate in some contexts.

**Figure 2:**
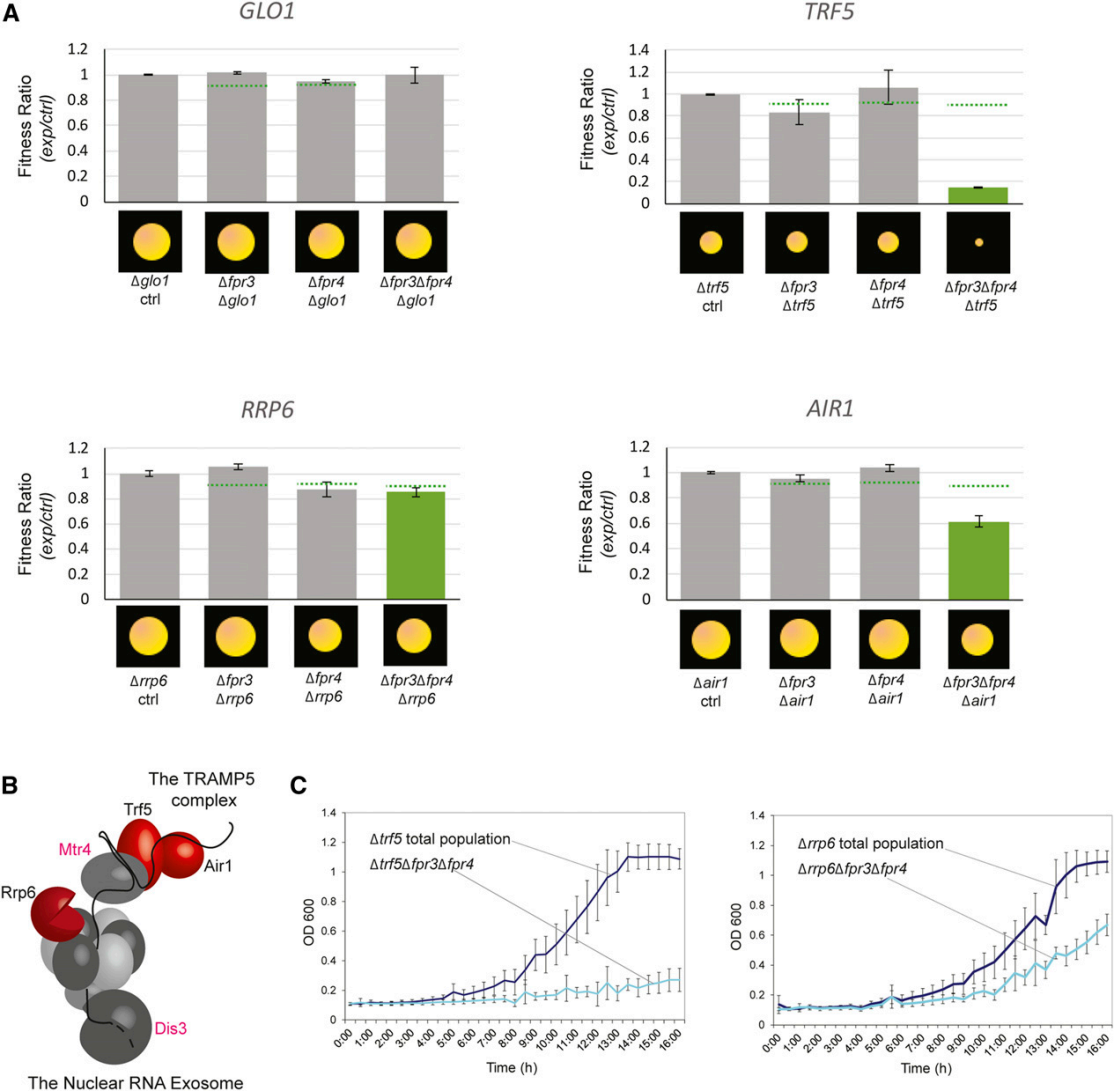
The TRAMP5 nuclear RNA exosome is a masked genetic interactor of *FPR3* and *FPR4*. (A) Fitness ratios of the indicated single, double, and triple mutants generated from paralog-SGA screens. The mean colony size ratios of ∆*fpr3*∆*xxx*, ∆*fpr4*∆*xxx*, and ∆*fpr3*∆*fpr4*∆*xxx* mutants relative to colony sizes of ∆*xxx* total haploid meiotic progeny are plotted as histograms. Ratios significantly below the default cut-off threshold (dotted green line) are indicated with green bars. A Balony software–generated image of the mean colony size of each mutant, normalized to the plate median colony size, is illustrated along the *x*-axis. *GLO1* is a negative control that does not display a genetic interaction in ∆*fpr3*∆, ∆*fpr4*, or ∆*fpr3*∆*fpr4* screens. (B) A schematic of the TRAMP5 complex (top right) interacting with the nuclear RNA exosome (bottom left). Genetic interactors identified in A are colored red. Pink text indicates essential components. Illustration is adapted from (Wolin *et al.* (2012)). (C) Growth curves for select triple deletion mutants and corresponding total haploid meiotic progeny control populations confirm the slow growth of ∆*fpr3*∆*fpr4*∆*trf5* and ∆*fpr3*∆*fpr4*∆*rrp6* mutants.

A total of 75 masked genetic interactions are only detectible in double Δ*fpr3*Δ*fpr4* mutants (Figure 1C, bottom). These genes are essential only when both paralogs are absent, and thus highlight processes in which Fpr3 and Fpr4 are redundant. Most notably these interactors include *TRF5* and *AIR1* (Figure 2A), two nonessential components of the TRAMP5 nuclear RNA exosome, an RNA surveillance factor that recognizes, polyadenylates, and degrades aberrant RNA transcripts (Figure 2B) (LaCava *et al.* 2005; Houseley and Tollervey 2008; San Paolo *et al.* 2009; Wery *et al.* 2009). An additional nonessential subunit of the nuclear RNA exosome (*RRP6**)* was at the threshold of significance, using default Balony settings (Figure 2A). We independently confirmed synthetic sickness of Δ*fpr3*Δ*fpr4* with Δ*trf5* and Δ*rrp6*, using growth curves (Figure 2C). Negative genetic interactions with three nonessential components of the TRAMP5 exosome strongly suggests that Fpr3 and Fpr4 have redundant biological functions likely involving the negative regulation of RNAs.

### Suppressor genetic interactions of *FPR3* and *FPR4*

**Figure 3:**
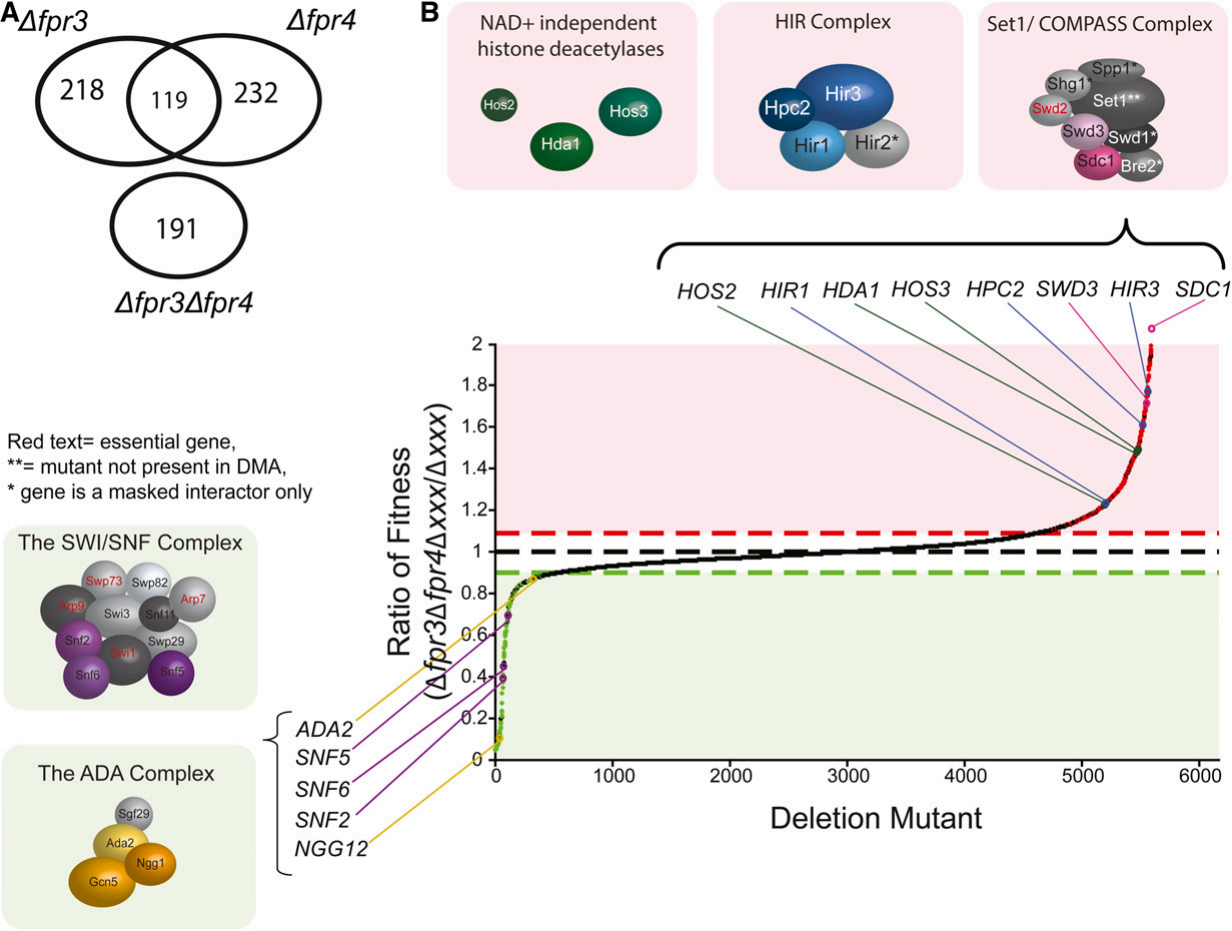
Suppressor genetic interactions support chromatin-centric functions for Fpr3 and Fpr4. (A) Venn diagram illustrating shared and unique suppressor interactors from ∆*fpr3* and ∆*fpr4* paralog-SGA screens. The number of suppressor genetic interactions only detectable in double deletion ∆*fpr3*∆*fpr4* mutants is represented below. (B) Plot of fitness ratios for all ∆*fpr3*∆*fpr4*∆*xxx* triple mutants relative to ∆*xxx* total haploid meiotic progeny controls. Green dots indicate negative genetic interactions, red dots indicate all suppressor genetic interactions. Threshold cut-offs are indicated by red and green dashed horizontal lines. The location of significant hits coding for components of chromatin modifiers are labeled and accompanied with schematic illustrations of their complex components. Components coded for by paralog-SGA hits are colored. Red text denotes essential complex components.

The SWI/SNF and ADA complexes are particularly important for the fitness of Δ*fpr3* and Δ*fpr4* yeast (Figure 1D). In support of a chromatin defect underlying these phenotypes, we found that several genetic suppressors (Figure 3) that alleviate the slow growth phenotype of Δ*fpr3*Δ*fpr4* yeast are themselves chromatin modifiers. These include Hos2, Hda1, and Hos3, three NAD^+^ independent histone deacetylases (*P* = 6.33 × 10^−5^); Hir1, Hpc2, and Hir3, three of the four components of the HIR replication-independent nucleosome assembly complex (*P* = 1.29 × 10^−5^); and Swd3 and Sdc1, two of the eight components of the Set1/COMPASS histone H3K4 methylase complex (*P* = 5.87 × 10^−3^). We note that the Swd2 subunit of COMPASS is encoded by an essential gene and the Δ*set1* knockout is not present in our deletion strain collection. It is particularly notable that we find histone deacetylases enriched among suppressor interactions and histone acetyltransferases among synthetic sick and lethal interactions. The presence of both aggravating and alleviating chromatin-related genetic interactions in our modified SGA screen is consistent with a chromatin-centric mode of action for Fpr3 and Fpr4.

### Fpr3 and Fpr4 regulate partially overlapping sets of genes

**Figure 4:**
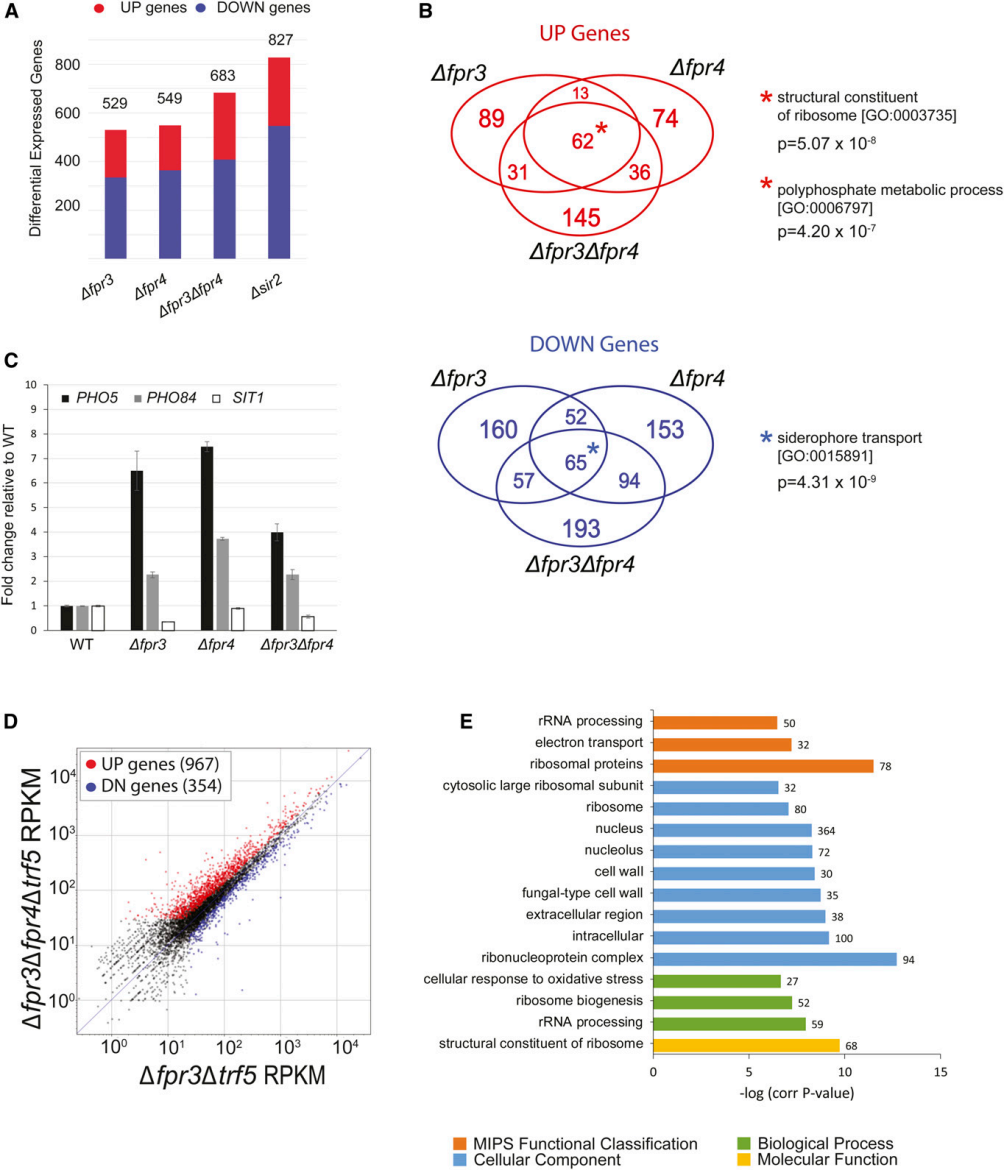
Fpr3 and Fpr4 have partially overlapping effects on the transcriptome. (A) Numbers of differentially expressed genes in ∆*fpr3*, ∆*fpr4*, ∆*fpr3*∆*fpr4*, and ∆*sir2* mutants. (B) Venn diagrams depict the partial overlap in up- and downregulated genes in ∆*fpr3*, ∆*fpr4*, and ∆*fpr3*∆*fpr4* mutants. Genes at the centers of the diagrams (*) are differentially expressed in all three RNA-seq data sets and are enriched in the indicated gene ontology terms. (C) Confirmation of select differentially expressed genes (*PHO5*, *PHO84*, and *SIT1*) by quantitative real-time PCR of RNA isolated from independent biological replicates. Fold changes in gene expression are shown relative to wild type. (D) Comparing the transcriptome of ∆*fpr3*∆*fpr4*∆*trf5* triple deletion mutants to ∆*fpr3*∆*trf5* double mutants reveals an increase number Fpr4-repressed RNAs (red dots). (E) Gene ontology enrichment analysis for upregulated transcripts in ∆*fpr3*∆*fpr4*∆*trf5* triple deletion mutants. Enriched genes were classified by molecular function, biological process, cellular component, and Munich Information Center for Protein Sequences (MIPS) functional database classification by FunSpec (http://funspec.med.utoronto.ca/). WT, wild type.

The genetic interactions of Fpr3 and Fpr4 with known chromatin modifiers suggest that they regulate transcription. Indeed a microarray study determined these histone chaperones regulate the expression of a broad set of functionally diverse protein coding genes (Park *et al.* 2014). Because these experiments did not include an analysis of Δ*fpr3*Δ*fpr4* double mutants and were restricted to protein coding regions of the genome, we sought to obtain a more complete view of the effects of Fpr3 and Fpr4 on the transcriptome. To this end, we performed a singlicate RNA-seq survey screen of the ribo-minus fraction of RNAs from wild-type, Δ*fpr3*, Δ*fpr4*, and Δ*fpr3*Δ*fpr4* yeast (Figure 4, A and B). To verify this survey approach, we included a Δ*sir2* strain as a control, which in our analysis displayed 854 differentially expressed genes (Figure 4A), using a lenient cut-off of 1.3-fold (a similar threshold to that of Park *et al.* (2014). The number and nature of Sir2-regulated genes we identified is in good agreement with previous reports of Sir2-regulated genes and binding sites (Li *et al.* 2013; Ellahi *et al.* 2015). A complete list of differential expressed genes from these experiments can be found in Appendix 3.

Single deletion mutants of Δ*fpr3* and Δ*fpr4* had 529 and 549 differentially expressed genes, respectively (Figure 4A, Appendix 3). Two general observations are consistent with previous microarray analyses (Park *et al.* 2014). First, roughly one-third of Fpr3-regulated transcripts are also regulated by Fpr4, and vice versa, confirming that on these genes, transcriptional regulation requires cooperation between paralogs (Figure 4B). Second, the effect of these histone chaperones on gene expression can be positive or negative, but the effect of Fpr3 and Fpr4 is always in the same direction. Since approximately two-thirds of differentially expressed genes were downregulated (Figure 4, A and B, blue), these histone chaperones appear to predominantly promote gene expression.

To determine if Fpr3 and Fpr4 have distinct effects on the transcriptome, we subjected the gene lists represented by sectors in Figure 4B to gene ontology analysis. While the singlicate nature of our comparative RNA-seq approach means the interpretation of DE genes should be taken with caution, it is noteworthy that genes uniquely regulated by Fpr3 and Fpr4 appear to fall into functionally distinct categories (Appendix 4). The 337 Fpr3-regulated genes retrieved the term transferase activity (*P* = 2.11 × 10^−7^) and the generic term of metabolic process (*P* = 1.99 × 10^−5^), while Fpr4-regulated genes are enriched in RNA-binding functions (*P* = 7.69 × 10^−6^), nucleotide-binding functions (*P* = 1.91 × 10^−5^), ribosome biogenesis processes (*P* = 1.07 × 10^−11^), and rRNA processing processes (*P* = 8.51 × 10^−9^). A total of 338 genes were uniquely misregulated in Δ*fpr3*Δ*fpr4* double mutants, but these genes generally fall into previously described Fpr3 and Fpr4 categories, including transferase activity (*P* = 4.45 × 10^−5^) and rRNA binding (*P* = 4.99 × 10^−4^). Taken together, these results indicate that Fpr3 and Fpr4 have nonoverlapping effects on a fraction of the transcriptome, but may be functionally redundant on some genes.

We identified 127 genes (62 upregulated, 65 downregulated) that are differentially expressed in all three RNA-seq libraries (Δ*fpr3*, Δ*fpr4*, and Δ*fpr3*Δ*fpr4*). Genes downregulated in all three experiments are enriched in factors involved in iron siderophore transport (*P* = 4.33 × 10^−9^). We found that the 62 upregulated genes are highly enriched in ribosomal protein genes (*P* = 5.07 × 10^−8^) and factors involved in phosphate transport (*P* = 1.24 × 10^−6^) and polyphosphate metabolism (*P* = 4.20 × 10^−7^). In fact, the most differentially expressed genes in our survey (up to 60-fold upregulated) are phosphate metabolic genes such as *PHO5* and *PHO11**/**12*, encoding acid phosphatases; and *PHO89*, *PHO84*, and *PIC2*, encoding phosphate transporters. Since previous studies did not identify the *PHO* genes as Fpr3/4-regulated, we verified our RNA-seq observations using independent biological replicates and quantitative real-time PCR of two *PHO* genes (Figure 4C), as well as one downregulated siderophore transporter, *SIT1*. The identification of polyphosphate metabolism and ribosomal protein genes as Fpr3/4 targets is noteworthy given a recent report that identified Fpr3 and Fpr4 as major direct targets of protein polyphosphorylation, and established conserved links between the polyphosphorylation and ribosome biogenesis network in yeast and human cells (Bentley-DeSousa *et al.* 2018).

In summary, our RNA-seq experiments demonstrate that Fpr3 and Fpr4 have nonoverlapping effects on the transcriptome. Most significantly, we find that both paralogs are required for repression of genes involved in phosphate uptake and polyphosphate metabolism, as well as ribosomal protein genes.

### The TRAMP5 RNA exosome masks the effects of Fpr4 on transcription

Deletion of *TRF5*, encoding the defining component of the TRAMP5 nuclear RNA exosome, induces severe sickness in Δ*fpr3*Δ*fpr4* yeast (Figure 2). We therefore wondered whether TRAMP5 might be required for the degradation of transcripts negatively regulated by these paralogs. To test this idea, we focused on Fpr4-regulated genes by sequencing the ribo-minus transcriptomes of two strains from our SGA screen: Δ*trf5* haploids with a functional Fpr4 (Δ*fpr3*Δ*trf5**)*, and isogenic haploids from the same spores that lack both Fpr3/4 proteins (Δ*fpr3*Δ*fpr4*Δ*trf5**)*. This provided a sensitized approach to reveal Fpr4-regulated RNAs because functional compensation by Fpr3 is not possible and potential degradation of upregulated RNAs by TRAMP5 is eliminated. This comparison (Δ*fpr3*Δ*trf5* *vs.* Δ*trf5*Δ*fpr3*Δ*trf5**)* uncovered a total of 1321 differentially expressed genes (967 upregulated and 354 downregulated) (Figure 4D). A summary of gene ontology analysis of upregulated genes is provided in Figure 4E. Genes encoding protein components of the cytosolic ribosome (*P* = 3.21 × 10^−12^) and genes associated with rRNA processing (*P* = 1.14 × 10^−8^) are highly enriched as Fpr4 targets. Also enriched were genes coding for constituents of the fungal-type cell wall (*P* = 1.87 × 10^−4^) and the electron transport chain (*P* = 6.12 × 10^−8^) (Figure 4E). These results partially explain the underestimation of genes negatively Δ*fpr4* transcriptomes (Figure 4A). That is, the TRAMP5 RNA exosome may buffer changes in the levels of some Fpr4 regulated RNAs.

### A signature of incomplete elongation is present in **Δ***fpr4* yeast

**Figure 5:**
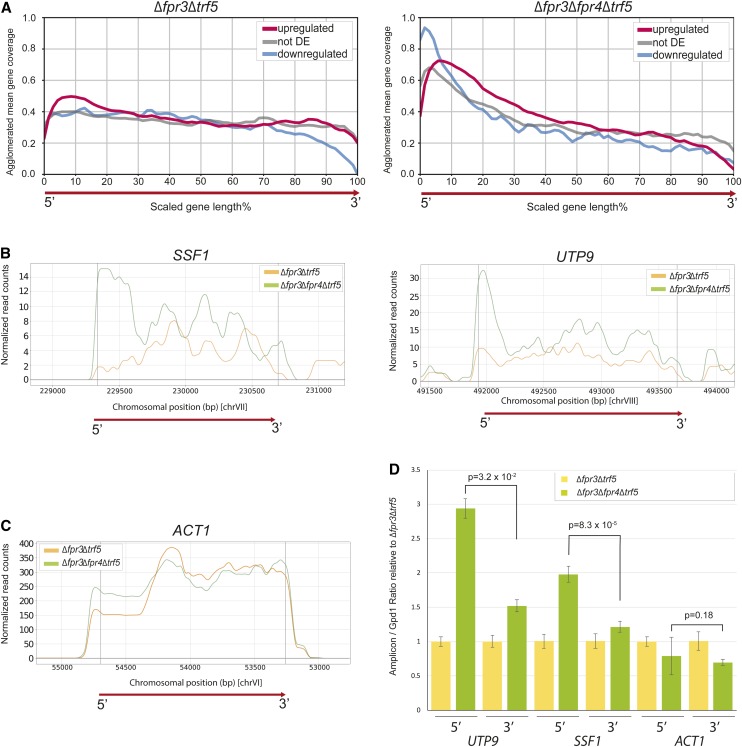
A signature of incomplete elongation is present in ∆*fpr4* yeast. (A) Plots of RNA-seq read density as a function of position on a scaled average gene. Upregulated, downregulated, and unchanged transcripts generated from (left) ∆*fpr3*∆*trf5* double mutants and (right) ∆*fpr3*∆*fpr4*∆*trf5* triple mutants are shown. (B) RNA-seq read density plots on two genes showing a signature of incomplete elongation: (left) *SSF1*, (right) *UTP9*. (C) RNA-seq read density plots on ACT1, a gene without a signature of incomplete elongation. (D) Quantitative real-time PCR validation of RNA read densities on *UTP9*, *SSF1*, and *ACT1*. 5′ and 3′ amplicons were normalized to the unchanged *GPD1* gene. RNAs were extracted from independent biological replicates (from those subjected to RNA-seq).

Further interrogation of our transcriptome data reveals additional evidence for Fpr4 in the regulation of transcription: we noticed that a significant proportion (∼40%) of differentially expressed genes in Δ*fpr3*Δ*fpr4*Δ*trf5* yeast displayed an accumulation of reads toward the 5′ end of the annotated transcript. Subsequent bioinformatic analysis of the total transcriptomes of Δ*fpr3*Δ*fpr4*Δ*trf5* and Δ*fpr3*Δ*trf5* mutants revealed that this asymmetry (or 5′-bias) is widespread, and detectable in genes, irrespective of their net change in transcription (Figure 5A). RNA-seq reads on two example genes illustrating this asymmetry signature are presented in Figure 5B; *SSF1* codes for a constituent of the 66S preribosome and is required for large ribosomal subunit maturation, while *UTP9* codes for a component required for proper endonucleolytic cleavage of 35S rRNA. The paired-end tag coverage on both of these genes, but not the *ACT1* gene (Figure 5C), displays the characteristic 5′ asymmetry in Δ*fpr3*Δ*fpr4*Δ*trf5* yeast. We verified these observations using independent biological replicates and quantitative real-time PCR using 5′ and 3′ amplicons of *UTP9* and *SSF1*, which were normalized to the unchanged *GPD1* gene (Figure 5D). This transcriptome signature demonstrates three novel findings: first, Fpr4 negatively regulates transcription from many genes even though total reads per gene may not change; second, Fpr4 action is critical at a stage after initiation, likely transcriptional elongation; and third, because this signature of accumulated 5′ reads on genes is only readily detectable in the absence of Trf5, the TRAMP5 RNA exosome can mask subtle transcriptional defects (Figure 4D). While we cannot rule out potential effects of Fpr3 and Fpr4 on the stability of RNAs, given the role histone chaperones play in nucleosome dynamics, we favor a model that explains this bias as a consequence of altered passage of polymerase through genes. Additional experiments probing transcriptional processivity in Δ*fpr3* and Δ*fpr4* mutant yeast are needed to resolve the mechanism(s) by which these histone chaperones facilitate the full transcription of genes.

### Fpr4 inhibits transcription from the nontranscribed spacers of rDNA

**Figure 6:**
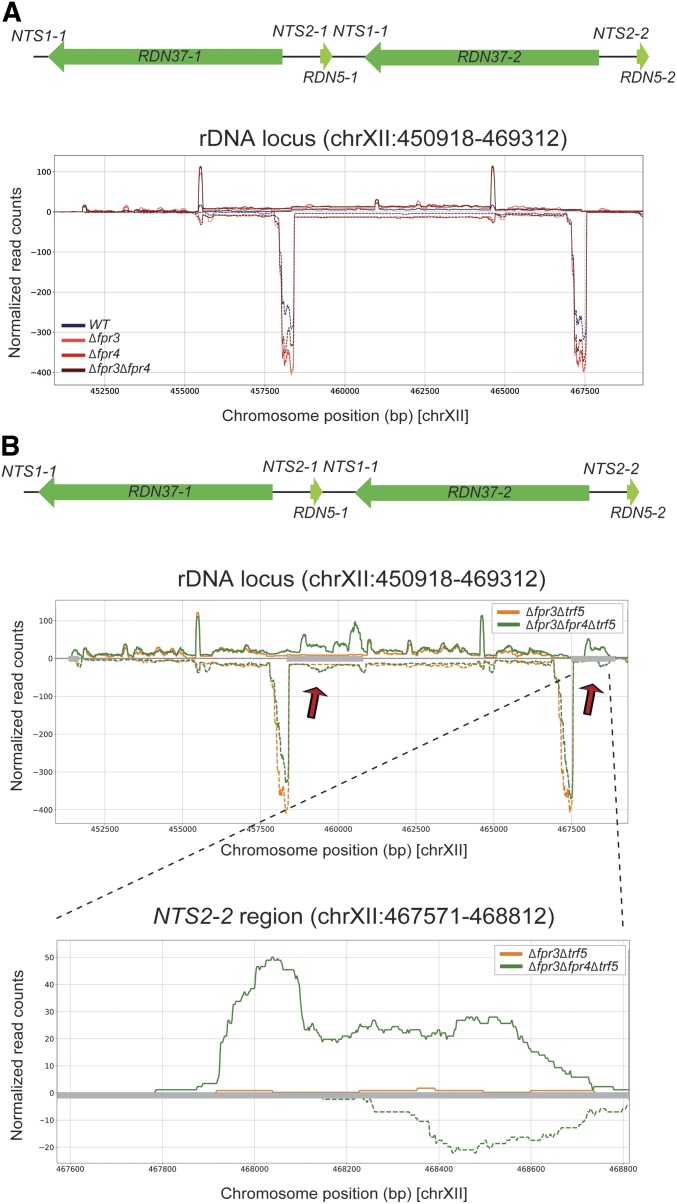
Fpr4 is required to silence the nontranscribed spacers (NTS) of rDNA. (A) Plots of RNA-seq read density across the rDNA locus on chromosome XII in wild type and ∆*fpr3*, ∆*fpr4*, and ∆*fpr3*∆*fpr4* mutants. The lack of reads mapping to NTS2-1, NTS1-1 (center) and NTS2-2 (right) suggests transcriptional silence in maintained in all strains. (B) Plots of RNA-seq read density across the rDNA locus on chromosome XII (top), and across NTS2-2 (bottom) in wild type and ∆*fpr3*∆*trf5* and ∆*fpr3*∆*fpr4*∆*trf5* mutants. The reads mapping to NTS2-1, NTS1-1 (center) and NTS2-2 (right) in ∆*fpr3*∆*fpr4*∆*trf5* reveals that Fpr4 is required to transcriptionally silence the NTSs. WT, wild type.

The rDNA locus in yeast consists of a series of 150–200 tandem repeats of a 9.1 kb unit containing the 35S and the 5S rRNAs, each separated by two nontranscribed spacer sequences (*NTS1* and *NTS2*) (Johnston *et al.* 1997). Given the nucleolar enrichment of Fpr3 and Fpr4, and the ability of Fpr4 to repress reporter expression from rDNA (Kuzuhara and Horikoshi 2004), we asked if yeast lacking Fpr3 and Fpr4 display transcriptional defects at rDNA. While our RNA-seq analysis was performed on ribo-minus RNA, reads from within the rRNA are readily detected (presumably from incomplete rRNA depletion) and indicate no change in rRNAs in Δ*fpr3*, Δ*fpr4*, or Δ*fpr3*Δ*fpr4* strains (Figure 6A), which we have also observed in Northern and quantitative real-time PCR analyses (data not shown). Surprisingly, we did not observe evidence for the reported loss of *NTS* silencing in Δ*fpr4* (or Δ*fpr3** or* Δ*fpr3*Δ*fpr4*) yeast (Kuzuhara and Horikoshi 2004) (Figure 6A). Given that TRAMP5 buffers the loss of Fpr4 (Figure 4D), we asked if Trf5 might be degrading NTS RNAs in Δ*fpr4* yeast. Consistent with this idea, we observe transcripts templated from both strands of NTS1 and NTS2 in Δ*fpr3*Δ*fpr4*Δ*trf5*, but not Δ*fpr3*Δ*trf5* strains. Taken together, these results support a model where Fpr4 establishes a transcriptionally silent chromatin state at rDNA. In the absence of this chromatin structure, pervasive transcription can occur from both strands of *NTS1* and *NTS2*. These RNAs are presumably normally degraded by TRAMP5.

### Fpr3 and Fpr4 are required for genomic stability at rDNA

Ribosomal RNAs comprise ∼80% of the total RNA in yeast; accordingly, active rDNA repeats are the most heavily transcribed and nucleosome-free genes in the cell (Warner 1999; Vogelauer *et al.* 2000; Nomura *et al.* 2004). Reciprocally, the adjacent *NTS* spacers and inactive rDNA repeats are chromatinized and potently silenced. This arrangement is thought to generate a chromatin template that is refractory to recombination between rDNA repeats and the deleterious loss of rDNAs from chromosome XII, which is a major driver of yeast replicative aging (Sinclair and Guarente 1997). For this reason, failure to generate heterochromatin environments at rDNA, as occurs in Δ*sir2* histone deacetylase mutants, decreases genomic stability at this locus (Gottlieb and Esposito 1989; Kobayashi *et al.* 2004).

**Figure 7:**
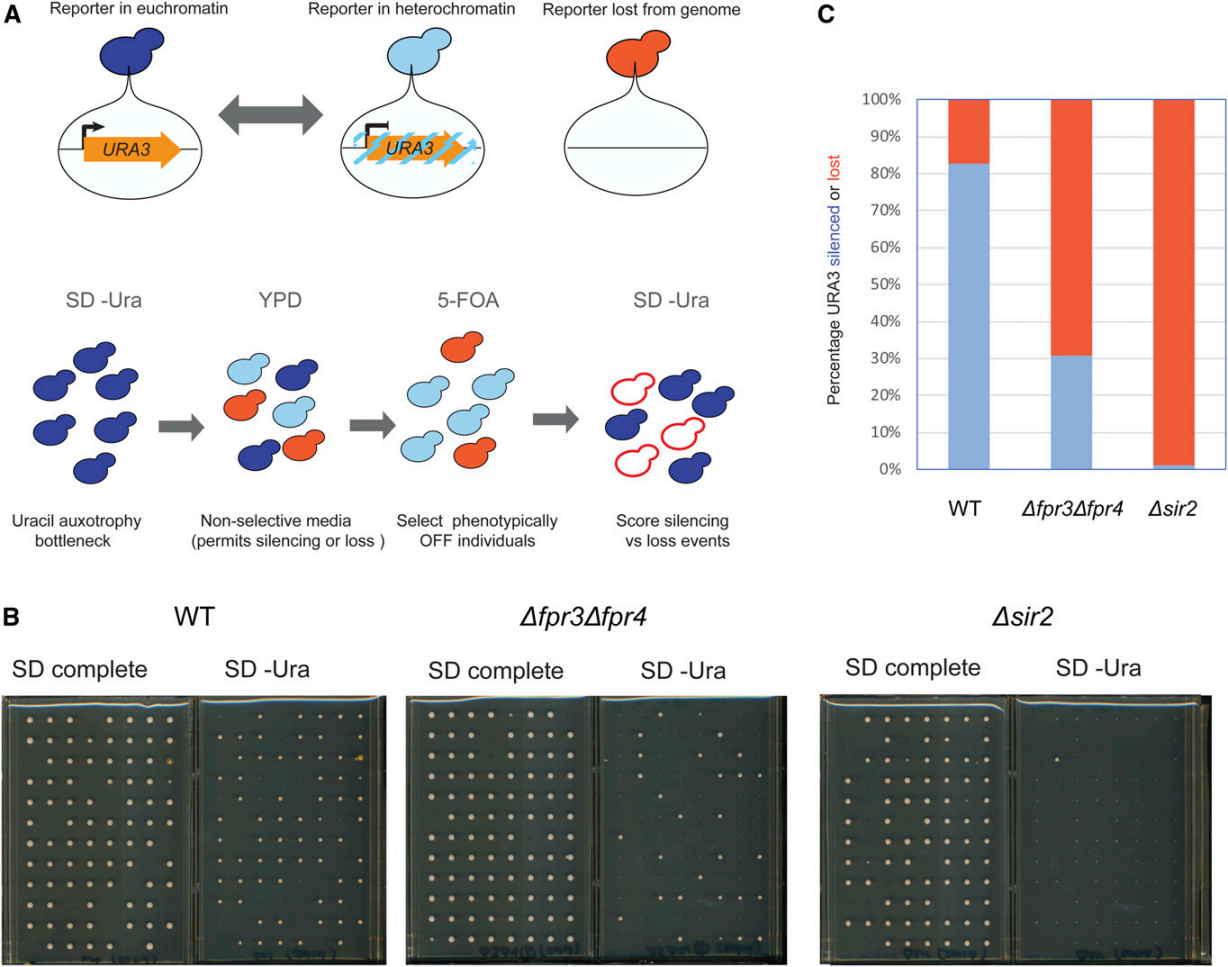
Fpr3 and Fpr4 are required for genomic stability at the rDNA locus. (A) Diagrams illustrating the propagation experiment carried out to assess frequency of reporter loss. Top: the rDNA(NTS1)::*URA3* gene stochastically switches between an active euchromatin state (dark blue cells) and a silenced heterochromatin-like state (light blue cells). Bottom: individuals that lose the reporter due to instability can be distinguished from cells with a stochastically silenced reporter with the indicated workflow. (B) Images of the 96 individuals selected for after propagation on SD complete control media and SD-URA experimental media. Those growing on the experimental media represent the fraction of the population in which the reporter was epigenetically silenced. Those that fail to grow indicate permanent loss of the reporter. (C) Percentage of total colonies recovered after strain propagation that have retained or lost the ability to grow on SD complete media. WT, wild type.

We reasoned that if Fpr3 or Fpr4 were silencing the NTS regions via a mechanism that involves chromatin structure, that yeast lacking these enzymes should also exhibit genomic instability at this locus. To test this hypothesis, we introduced Δ*fpr3*Δ*fpr4** and* Δ*sir2* deletions into a strain with a reporter gene (*URA3*) integrated at NTS1 (van Leeuwen and Gottschling 2002; van Leeuwen *et al.* 2002). First, URA^+^ status of each strain was ensured by propagation in media lacking uracil. Next, cells were grown in nonselective media (YPD) for 2 days to permit reporter silencing or loss. Phenotypically *ura*^–^ cells were isolated on 5-FOA and ∼96 colonies were picked using a colony picking robot. These *ura*^–^ cells could arise in two ways: epigenetic silencing of *URA3* at *NTS1*, or from *URA3* gene loss via recombination (Figure 7A). To discriminate between these events, we replica-plated these individual isolates to media lacking uracil, where growth indicates that the *URA3* phenotype was a consequence of epigenetic silencing. Reciprocally, isolates that failed to grow would represent reporter loss events (Figure 7A). These propagation assays revealed that normally, the rate of epigenetic switching of *URA3* is much higher than reporter loss: 82% of *ura*^–^ isolates still have a *URA3* gene at the end of our propagation assay as exemplified growth in the absence of uracil (Figure 7, B and C), and by PCR of genomic DNA (not shown). As expected, Δ*sir2* yeast are unable to establish silent chromatin at NTS1, and can only grow on 5-FOA via loss of the reporter. Finally, we observe that Δ*fpr3*Δ*fpr4* yeast are compromised in their ability to silence *URA3* epigenetically: only 30% of 5-FOA-resistant colonies retain the *URA3* gene. Thus, in Δ*fpr3*Δ*fpr4* yeast recombination and *URA3* reporter gene loss are more frequent than epigenetic silencing. This observation supports a model where Fpr3 and Fpr4 build chromatin structures at the *NTS* regions of rDNA locus. These structures are critical to maintaining genome stability at rDNA.

## Discussion

Gene duplication events play a critical role in protein and organism evolution. However, the high similarity of duplicated genes can lead to complete or partial compensation when one paralog is deleted, as is in the case in conventional genetic interaction analysis. Here we present a dual-query SGA screening approach where one genetic cross can report the separate, shared, and masked genetic interactions of gene paralogs. Using this approach on two nucleoplasmin-like histone chaperones revealed that they perform separate, cooperative, and redundant chromatin-related functions. Given that ∼13% of yeast protein coding genes are duplicates (Wolfe and Shields 1997), this approach may have applications in the analysis of other paralogs.

The genetic interactions annotated here support a unique function for Fpr3 in orchestrating centromeric chromatin dynamics during chromosome segregation. This is fully consistent with existing literature (Hochwagen *et al.* 2005; Krogan *et al.* 2006; Macqueen and Roeder 2009; Ghosh and Cannon 2013; Ohkuni *et al.* 2014). Our comparative analysis provides additional systems-level evidence that this role is not shared with Fpr4, indicating that Fpr3, potentially as a homo-oligomer, may regulate chromatin in a way that affects chromosome segregation (Hochwagen *et al.* 2005; Macqueen and Roeder 2009). Furthermore, the fact that Δ*fpr3*Δ*fpr4* double mutants display fewer genetic interactions than single-gene Δ*fpr3* mutants (Appendix 1) indicates that Fpr4 may be toxic in the absence of Fpr3 (Ohkuni *et al.* 2014). This model predicts that in the absence of Fpr3, the partial engagement or modification of chromatin by Fpr4 is deleterious.

Several members of the ADA and SWI/SNF chromatin regulatory complexes exhibit negative genetic interactions with both Fpr3 and Fpr4. These results could be explained by reduced dosage of a histone chaperone activity. Alternately, these genetic interactions are consistent with a model where Fpr3 and Fpr4 act together to chaperone nucleosomes, facilitating chromatin dynamics as SWI/SNF does. Whether this means that the paralogs operate together in a sequence of events, such as the removal and subsequent redeposition of nucleosomes during transcription or, in concert as a hetero-oligomeric complex, is not yet clear. The fact that Fpr3 and Fpr4 copurify (Krogan *et al.* 2006) supports the latter model, but does not exclude the former.

The repression of several phosphate and polyphosphate metabolism genes in rich media requires both Fpr3 and Fpr4. It is therefore intriguing that both Fpr3 and Fpr4 were recently identified as two of the most heavily polyphosphorylated proteins in the yeast proteome, along with several proteins in an evolutionarily conserved network of ribosome biogenesis factors (Neef and Kladde 2003; Bentley-DeSousa *et al.* 2018). The precise sites of Fpr3 and Fpr4 polyphosphorylation and the effect of this post-translational modification on Fpr3 and Fpr4 function is not yet clear. Fpr3 and Fpr4 also affect the steady-state levels of mRNAs encoding ribosomal protein genes and rRNA processing machinery. Thus, Fpr3 and Fpr4 may function as master regulators of ribosome biogenesis by coordinating both ribosomal protein abundance and rRNA processing. Given that many ribosomal and rRNA processing protein genes are driven by common regulators, Fpr3 and Fpr4 may recognize common DNA sequences or transcription factors to accomplish this function (Fermi *et al.* 2016). As already stated, the links between polyphosphorylation of Fpr3 and Fpr4 and the ribosome biogenesis network also require further investigation. It appears that at least some elements of this regulatory system may be conserved in the human nuclear FKBP25 protein (Gudavicius *et al.* 2014; Dilworth *et al.* 2017) and the acidic tract–containing nucleolin protein (Bentley-DeSousa *et al.* 2018).

The yeast TRAMP5 complex recognizes and polyadenylates aberrant RNA transcripts to target them for degradation by the Rrp6 ribonuclease (Schmidt and Butler 2013). TRAMP5 targets include both ribosomal protein coding mRNAs and cryptic unstable transcripts generated from intragenic sites on the genome, including those within the rDNA locus (LaCava *et al.* 2005; Reis and Campbell 2007; San Paolo *et al.* 2009; Wery *et al.* 2009). Here, we found that deletion of Δ*trf5* enabled the detection of a previously invisible transcriptome signature Δ*fpr4* yeast where there is a bias in the RNA-seq reads toward the 5′ end of genes. This is consistent with Fpr4 promoting the transcriptional elongation process. It is noteworthy that these reads appear to cover the first one to three nucleosomes of genes because Fpr4 is capable of both histone and nucleosome binding (Leung *et al.* 2017), and was previously shown to be important for the kinetics of transcriptional induction (Nelson *et al.* 2006). Thus, the nucleosomes near the transcriptional start site are candidates targets of Fpr4. This regulation could involve either the installation of nucleosomes within promoters to inhibit transcriptional initiation or nucleosome/histone eviction from sequences downstream of the promoter to remove nucleosome blocks to the polymerase. The cryo-electron microscopy structures of nucleoplasmin pentamers engaging intact histone octamers provides further support for these models (Franco *et al.* 2019). We recently showed that Fpr4’s nucleoplasmin-like acidic regions bind to free histones, while its basic surfaces permit nucleosome binding (Leung *et al.* 2017). Precisely how these activities and Fpr4’s peptidyl-prolyl isomerase activity toward the histone H3 tail (Nelson *et al.* 2006) (Monneau *et al.* 2013) cooperate to regulate chromatin dynamics is still unclear, However, the genetic and transcriptional readouts identified here provide complementary assays for dissecting the importance of each of these features.

In addition to regulating the transcription of protein coding genes, Fpr4 restricts transcription from the *NTS* sequences of rDNA. This is consistent with both nucleolar enrichment and data indicating that Fpr4 inhibits transcription of exogenous reporters at rDNA in yeast (Kuzuhara and Horikoshi 2004) and orthologs operate similarly in plants (Li and Luan 2010). In yeast, the NTS loci contain important DNA sequence features, including two terminators for the RNA Pol I–transcribed RDN35 repeat, a replication fork barrier site, and an autonomous replication site. Two separate observations suggest that Fpr4 builds chromatin at rDNA to insulate DNA at these spacers. First, using a strain sensitized to reveal Fpr4-regulated RNAs accumulates large amounts of NTS transcripts, and these RNAs are templated by both DNA strands. Second, consistent with a chromatin structural defect underpinning this phenomenon, the rDNA locus in Δ*fpr3*Δ*fpr4* yeast is also hyper-recombinogenic (Figure 7). Thus, these histone chaperones are of particular importance at the 100–200 rRNA repeats where they mediate the stability and silencing of spacers between the most heavily transcribed sequences in the cell. How these chaperones regulate chromatin structure at this locus, and how the structure differs from other targets in the nuclear genome, remain open questions that can now be addressed in future studies.
